# Evaluation of exhaust emissions of agricultural tractors using portable emissions measurement system in Korean paddy field

**DOI:** 10.1038/s41598-024-53995-0

**Published:** 2024-02-12

**Authors:** Wan-Soo Kim, Seung-Min Baek, Seung-Yun Baek, Hyeon-Ho Jeon, Md. Abu Ayub Siddique, Taek-Jin Kim, Ryu-Gap Lim, Yong-Joo Kim

**Affiliations:** 1 Department of Bio-Industrial Machinery Engineering, Kyungpook National University, Daegu, 41566 Republic of Korea; 2 Upland Field Machinery Research Center, Kyungpook National University, Daegu, 41566 Republic of Korea; 3 Department of Smart Agriculture Systems, Chungnam National University, Daejeon, 34134 Republic of Korea; 4 Department of Biosystems Machinery Engineering, Chungnam National University, Daejeon, 34134 Republic of Korea; 5 Department of Drive System Team, TYM R&D Center, Iksan, 54576 Republic of Korea; 6 Department of Smart Agriculture, Korea Agriculture Technology Promotion Agency, Iksan, 54667 Republic of Korea

**Keywords:** Environmental sciences, Engineering

## Abstract

Recently, diesel engine emissions have been designated as a first-class carcinogen by the World Health Organization (WHO). As such, problems with diesel engine emissions continue to increase around the world. This study aimed to analyze the emissions (CO, NOx, PM) of agricultural tractors during farming operations in order to build a reliable national inventory of air pollutant emissions. Emission data were collected using a portable emission measurement system during actual agricultural operation. The load factor (LF) of the engine was calculated using the collected engine information, the emission factor was analyzed using the LF and the measured emission. The LF was significantly different from the current standard value of 0.48, which is used in Korea to calculate exhaust emissions. The deviation ratio of the emission factor was 0.039 ~ 56.59 compared to Tier-4 emission regulation standards. Under many conditions, the calculated emission factor was higher than the emission limit. Thus, this study provides useful information for emission inventory construction through emission calculation under actual conditions and suggests the need to realize the currently applied emission factor.

## Introduction

Recently, World Health Organization (WHO) designated diesel engine exhaust as a class 1 carcinogen. As such, issues related to diesel engine exhaust continue to increase worldwide, and various environmental regulations are emerging to resolve this issue^1^. In particular, air pollution from non-road mobile machinery (NRMM), including construction and agricultural machinery, has increased significantly in many countries worldwide^2^. The proportion of air pollutant emissions by non-road mobile pollution sources (NRMPSs), including railways, ships, aviation, construction machinery, and agricultural machinery, which represent a major emission source category, in Korea has gradually increased from 11.9 in 2013 to 25.3% in 2017^3^. In particular, a large proportion of the domestic air pollutant emissions was due to NRMPSs, accounting for approximately 22% of the carbon monoxide (CO) and 26% of the nitrogen oxides (NOx) in the total air pollutants from Korea in 2017^4^.

Because the tractor can be used universally for various agricultural tasks, it is considered a representative agricultural machine type^5^. In particular, tractors have the highest working area among major agricultural machinery in Korea, with a working area of 21.7 ha/unit and 35.6 working days per year^6^. The number of tractors owned in Korea continues to increase from 264,834 ea in 2010 to 302,570 ea in 2020^6^. Tractors perform agricultural work by towing or supplying power to the attached implement during operation^7^. In Korea, tractors are used 54.9% of the time for tillage, grading and leveling operations^6^. Consequently, their operating environments are harsh, with severe load fluctuations, which directly affects exhaust emissions^8,9^.

Air pollutant emissions in Korea are calculated using the clean air policy support system (CAPSS) of the national air emission inventory and research center (NAIR) of the Ministry of Environment^10^. The emission data of air pollutants from emission sources are important for establishing national air pollutant management policies^11^. These policies will be closely related to environmental and energy loads as well as people's health issues. However, the emissions of agricultural machinery currently managed by CAPSS are calculated using the number of agricultural machinery, load factors (LFs), etc., based on emission factors developed by the US environmental protection agency (EPA)^3^. Because the agricultural work environment and conditions (especially soil) in Korea are very different, the accuracy of this method low, and the reliability of the data cannot be easily secured. It is also impossible to calculate the amount of emission that reflects actual working conditions^12^. In addition, because the currently applied emission factor was calculated for an engine unit using a dynamometer, applying it to reflect the actual agricultural work conditions is difficult. To overcome this problem, in the US and Europe, vehicle monitoring is performed using a portable emission measurement system (PEMS)^13–15^. In the field of construction machinery, classified as NRMM, along with agricultural machinery, some studies have reported the measurement of real working emission (RWE) data under actual vehicle conditions using a PEMS^17–20^. Kim and Lee measured exhaust emission data using a PEMS under actual working conditions (no load and load conditions) of an excavator and analyzed the correlation between engine load and major factors of exhaust emissions to estimate CO_2_ emissions^16^.

In the field of agricultural machinery, some studies on the measurement of exhaust gas emissions from tractors using PEMS equipment have been conducted by researchers from countries in the United States and Europe^2,21^. Lijewski and Merkisz, in which the emissions of passenger vehicles and agricultural tractors were compared based on actual driving under on-road conditions^11^. They reported that the emissions of air pollutants (CO_2_, CO, NO_x_, HC, and PM) for tractors were higher than those for passenger vehicles. In particular, the largest differences were recorded for road emissions of CO and NO_x_ (90 and 97% lower, respectively, for passenger cars). Merkisz et al established a measurement system using a PEMS to measure the CO_2_ emissions of tractors according to actual vehicle conditions and conducted experiments at three speeds (5, 10, and 15 km/h)^21^. It was reported that the CO_2_ emissions per unit area at 10 km/h were the highest (18.8 kg/ha). Lindgren and Hansson simulated the effects of engine control strategies and transmission characteristics on the exhaust gas emissions of agricultural tractors according to on-road transport and soil cultivation^22^. They reported that different driving strategies and transmission characteristics can be used to significantly influence emissions without affecting work hours or fuel consumption. However, in Korea, there has been no case of measuring RWE using PEMS under actual vehicle conditions, and only a few studies have been reported in which exhaust emissions were estimated using fuel consumption^11,22^. Therefore, research is required to analyze the exhaust emissions and emission factors of each air pollutant in Korea by measuring the tractor RWE generated under actual working conditions^10,23^.

The aim of this study is to secure basic data and evaluate standard rationalization for emission factors. To this end, in this study, the engine characteristics and exhaust gas emissions of agricultural tractors were measured and analyzed according to various tillage treatments (moldboard plow tillage and rotary tillage operations).The detailed research goals are as follows: (1) to develop a data measurement system for measuring tractor engine characteristics and exhaust emissions; (2) measure and analyze tractor engine and exhaust emission data through actual tillage operations; (3) map the measured engine characteristics using the actual work on the engine performance curve; and (4) evaluate emission factors by comparing the analysis results of emission factors with current emission regulations.

## Methods

### Test engine

In this study, a four-wheel drive tractor was used to measure engine characteristics and exhaust emissions during actual field operation. The dimensions and empty weight of the tractor were 4020(L) × 2270(W) × 2790(H) mm and 4000 kg, respectively. The maximum traction force of the tractor was 26.18 kN at a travel speed of 2.08 km/h, and the maximum running speed was 33 km/h. The tractor used is a 2019 model, and the engine mounted on the tractor under test was a diesel engine that satisfied Tier-4 emission regulations. The engine displacement was 3409 cc and the compression ratio was 17:1. The engine rated torque and power of the tractor were 290 Nm and 67 kW, respectively, at the rated engine rotational speed of 2200 rpm. The tractor was equipped with selective catalyst reduction (SCR).

### Measurement system

**Figure 1:**
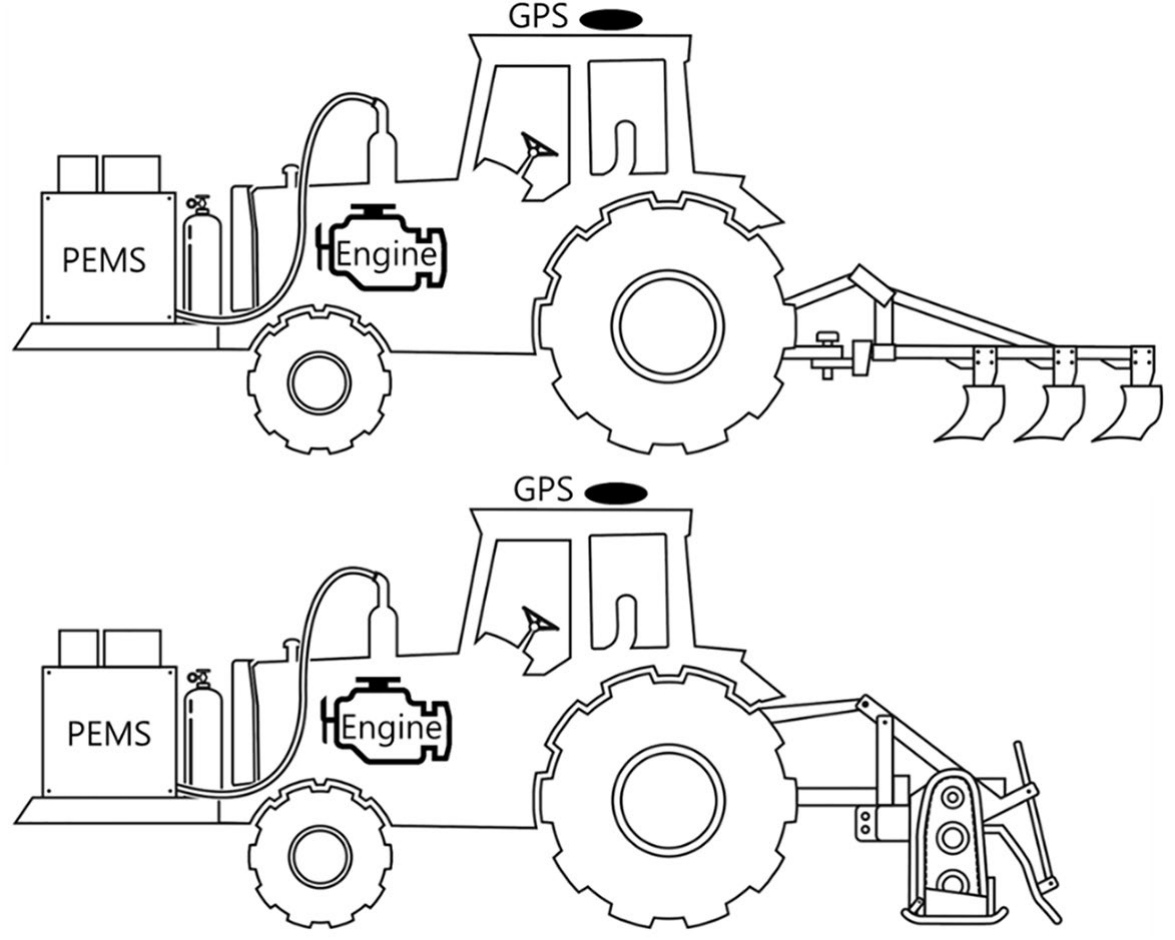
Measurement tractor layout equipped with sensor system. Engine = Engine properties (torque, speed, and power) and fuel consumption; GPS = Travel speed; and PEMS = CO, NOx, PM, and exhaust flow rate.

A tractor measurement system was constructed to measure the engine characteristics and exhaust emissions according to the tillage operations, as shown in Fig. 1. Engine characteristics such as torque, rotational speed, and power and fuel consumption of the tractor were collected in real time through controller area network (CAN) communication according to the J1939 protocol. In this study, a PEMS was used to collect tractor exhaust emission data using the RWE during the major tillage operations. The PEMS (OBS-one, Horiba, Kyoto, Japan) used in this study is an on-board exhaust gas measurement system used in various industrial fields, such as automobiles, construction machinery, and agricultural machinery. It can measure exhaust volume flow rate (EVFR), CO, NOx, PM, etc^15,24^. This PEMS is divided into gas analyzer (CO, NOx) and particle analyzer (PM). In the emission gas calculation, non-dispersive infrared (NDIR); a heated chemiluminescence detector (HCLD); and the filter gravimetric method (FGM), diffusion filling method, and diffusion charging method (DCM) were used for CO; NOx; and PM, respectively. The PEMS used in this study applies a dilution sampling method, and the dilution ratio is 10–20:1. The information measured by a gas analyzer is measured in dry form by removing moisture from the sample before measurement, and then converted to wet form through post-processing. For particle analyzers, measurements are made in real time in μg/m^3^. Therefore, in the case of PM, the separate dry–wet concept is not applied. The temperature of the filter block is maintained at 40–50 °C while the equipment is operating. One hour before/after the test, the PM filter is conditioned for a certain period of time under constant temperature/humidity conditions and the weight is recorded. The exhaust gas temperature is measured in the PEMS module, not the engine, after passing through a pipe of 2.5 m, so the results are expected to be slightly lower than the engine temperature. The PM sensor has its own zero-point adjustment function, and the equipment was calibrated to zero before and after the test. In accordance with RDE (real driving emission) regulations, the PEMS equipment was calibrated (zeroing and spanning) using standard gases before and after the RDE test, which lasted approximately 4 h. The standard used for calibration is a product of Daewoo Gas Corporation, and the concentrations of the span calibration gas are 7690, 1540, and 259.6 μmol/mol for CO, NO, and NO_2_, respectively. Sensor drift was confirmed through zeroing and spanning calibration before and after testing. The system response time of PEMS components is less than 12 s. The time-alignment of data collected from various sensors (Exhaust gas, GPS, engine OBD) was matched by taking into account operation start and end times. The PEMS system used in this study includes data analysis software with a built-in time alignment function, which solves the problem of response time differences between various components of PEMS. The detailed specifications and calculation method for the emission gas of the PEMS are listed in Table 1.

**Table 1:** Specification of the PEMS for measuring exhaust emissions of the tractor.

Items		Method	Range	Acc
Exhaust flow rate		Pitot flow meter	18.9~809.8 kg/h (100 $^\circ{\rm C}$) 28.4~602.9 kg/h (400 $^\circ{\rm C}$)	± 2.0%
GAS	CO	NDIR	0~8%	
	NOx	HCLD	NO: 0~3000 ppm–NO_2_: 0~1000 ppm	
PM		FGM&DCM	23 nm~2.5um (Particle size)	

**Figure 2:**
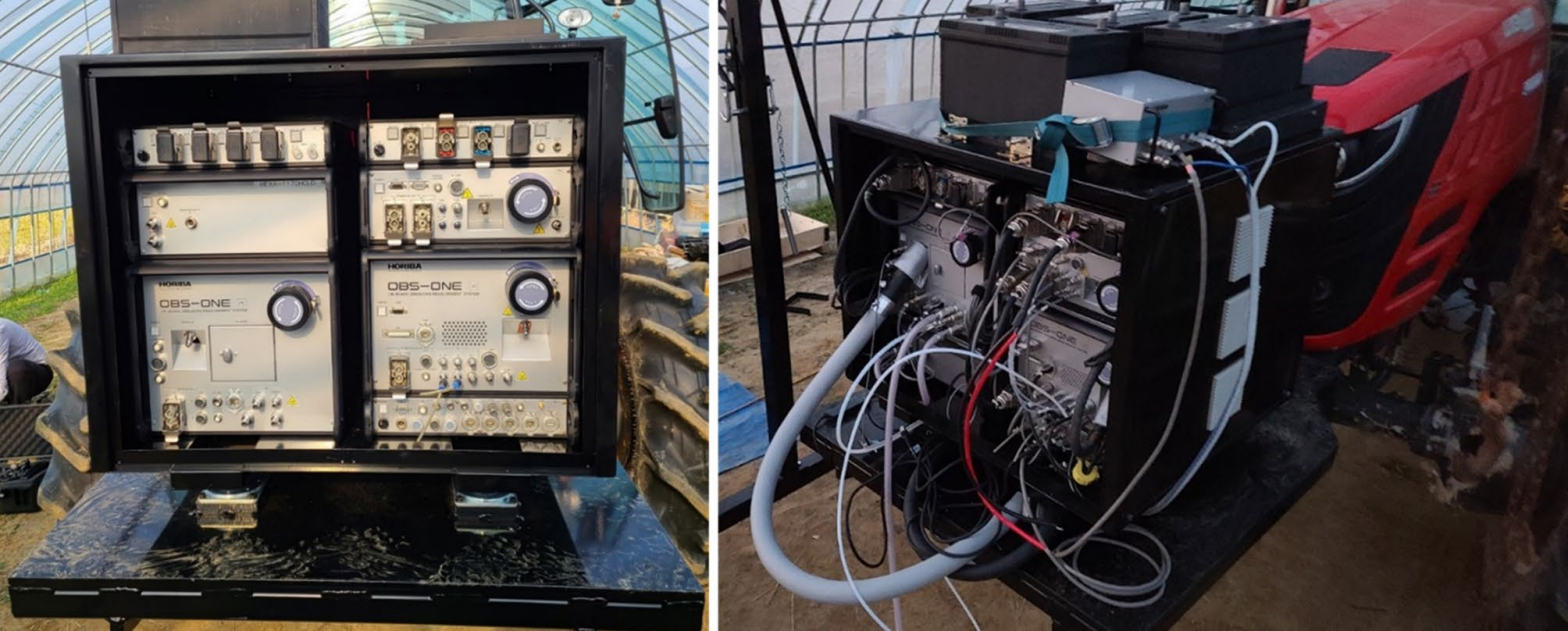
Portable emissions measurement system attached to the front part of the tractor.

The PEMS equipment was covered using a casing jig to protect it from the dust generated during agricultural work. There was insufficient space to install the PEMS on the tractor; therefore, the existing ballast was removed, and the PEMS was installed in the ballast position in front of the tractor, as shown in Fig. 2. The weights of the PEMS and jig were approximately 100 and 200 kg, respectively, and the total added weight was 300 kg (Table 2).

**Table 2:** Specifications of the implements used for tillage operations.

Items	Moldboard plow	Rotavator
Model	WJSP-8	E260
Length × width × height (mm)	2150 × 2800 × 1250	860 × 2760 × 700
Weight (kg)	790	715
Furrows/blades	8	60
Working width (mm)	2800	2580
Required power (kW)	65–90	65–90

### Field experiment

The field experiment was conducted in October 2020 in a paddy field of 3132 m^2^ (36 m $\times$ 87 m) located at 674–10, Dangsan-ri, Dangjin-si, Chungcheongnam-do, South Korea (36°56' 04.0" N 126°37' 58.1" E). The ambient temperature and humidity of the field experiment site were 17 to 20 ℃ and approximately 75%, respectively. The experiment was conducted for approximately 4 h per day over the entire field experiment site. The soil texture of the field experiment site was Loam by the soil classification triangle of United States Department of Agriculture (USDA), and the soil moisture content was measured at 20 random locations in the test sites using soil moisture sensor (TDR350; Spectrum Technology, Aurora, IL, USA), and the average value was 41.8%. Plow tillage and rotary tillage, which are the most widely used major tillage operations in Korea, were selected for field data collection. The implements used were a moldboard plow (WJSP-8, Woongin Machinery Co., Ltd., Gimje, Korea) and rotavator (E260, Celli SpA, Forli, Italy)^25^. The depth during tillage operations was set to be maintained at the 15–20 cm level according to the recommendations of farmers in consideration of the characteristics of agricultural operations in Korea. The number of working stages was selected as B3 (7.60 km/h) for plow tillage and A3 (2.67 km/h) for rotary tillage^26^. The data from the tillage operations used in this study were based on the minimum unit condition consisting of one set of straight forward (tillage) and steering operation. The agricultural operation of the tractor was carried out in a C-type pattern. The engine rotational speed was set at the rated speed (2300 rpm). Tractors are controlled by decreasing engine rotational speed (lowering the throttle) and increasing torque when higher torque is required based on real-time agricultural work load. Therefore, basically the tractor is operated at the 2300 rpm, but when there is a demand for a high load, the engine rotation speed may be lowered. In this study, only data for hot conditions after the tractor's engine was sufficiently preheated were used for analysis, and data on cold conditions were not considered. To collect data only after the engine was sufficiently hot, the experiment was performed 5 min after engine start. This is a result that also satisfies the values presented in previous studies^27^. The reference value of the cold condition (cold start) was based on the coolant temperature of less than 70°C as defined in EU Directive 2012/46/EU, and temperatures above that were considered hot condition^28^.

Tractors perform tillage operations by traveling straight ahead, but they also turn at the end of the straight path to work on the next row. The characteristics of the tractor's load and exhaust emission are different for tillage operation at straight path and steering operation at turning work sections. Therefore, in this study, the entire work section of the tractor was divided into a tillage section and a steering section. Depending on the operating conditions, the dynamic characteristics of the engine vary significantly, which directly affects the exhaust emissions^29^. Therefore, in this study, the data collected during the two tillage operations were divided into tillage and steering sections, respectively, and the dataset for each section was analyzed^10,11^. The sampling rate for both tillage operations is 200 Hz. The data collection times for plow and rotary tillage operations were 117.91 and 142.89 s, respectively. The number of data used in the analysis was 13,588 and 9994, respectively, for tillage and steering operations in plow tillage and 20,758 and 7820, respectively, for tillage and steering operations in rotary tillage.

### Data analysis

#### Load factor

The LF refers to the average power ratio of the engine; it is an important indicator that shows how much power is actually used compared to the rated power of the engine and is significant for calculating the exhaust emission factors of air pollutants and emission sources^30^. In Korea, the LF of agricultural machinery and construction machinery is collectively applied as 0.48, regardless of conditions such as type, model, and year of machinery^4^. Because this does not reflect the engine load characteristics that vary depending on various conditions, a method that reflects the actual LF is necessary. In this study, real-time engine power was measured using Eq. (1) based on engine rotational speed and torque data measured according to actual agricultural operations, and LF was derived using Eq. (2) using real-time measured engine power and the rated power.

1 $${\text{EP}}=\frac{2\pi TN}{\mathrm{60,000}},$$

2 $${\text{LF}}=\frac{{EP}_{a}}{{EP}_{r}},$$

where ${\text{T}}$ denotes torque (Nm), ${\text{N}}$ denotes rotational speed (rpm), ${\text{EP}}$ denotes the engine power (kW), ${EP}_{a}$ denotes measured engine power and ${EP}_{r}$ denotes rated engine power.

### Data analysis

#### Exhaust emission and emission factor

In Korea, the emissions from agricultural machinery, including tractors, are calculated and managed by NAIR through CAPSS^4^. In CAPSS, emissions are calculated using the number of units, engine rated power, LF, annual operating time, and emission factor based on Eq. (2)^4^. The annual agricultural machinery yearbook published by the Korean Society of Agricultural Machinery (KSAM) was used to calculate the number of tractors^6^, and the results of a survey on agricultural machine use, provided by the National Institute of Agricultural Sciences (NAS) of the Korea Rural Development Administration, were used to calculate the annual operating time^31^. As mentioned in Sect. (“Load factor”) the LF of 0.48 is currently applied collectively; however, in this study, the value calculated using Eq. (1) was applied. The emission factor of the tractor from the Korea National Air Pollutant Emissions Guidebook (IV) published by NAIR was adopted^4^. In Korea’s national air pollutant emissions inventory guidebook (IV), emission factors are classified into those before 2012 (~Tier-2), those between 2013–2014 (Tier-3), and those after 2015 (Tier-4) according to environment regulations, as shown in Table 3. In this study, the emission factor according to the RWE was calculated using Eq. (3), and the calculated emission factor for Tier-4 regulation, listed in Table 3, were compared and evaluated. Because this study analyzed the emission factor using the real-time LF and exhaust emissions for a single tractor, the number of tractors and annual operating time were not taken into account.

3 $$E=\sum \frac{N\times LF\times EP\times HRS\times EF}{{10}^{3}},$$

where $E$ denotes the exhaust emission (kg/year), $N$ denotes the number of units, $LF$ denotes the load factor, $EP$ denotes the engine rated power (kW), $HRS$ denotes the annual operating time (h/year), and $EF$ denotes the emission factor (g/kWh).

**Table 3:** Emission factor of agricultural machinery according to air pollutant based on the regulation stages.

Regulation stages	Applicable model year	Emission factor (g/kWh)^a^		
		CO	NOx	PM
Tier-4	2015~	0.071	0.188	0.016

^a^The emission factor depends on the engine power, and the above values are based on a 67-kW engine.

#### Evaluation

To analyze the standard deviation with respect to the mean of the sample group, the relative standard deviation (RSD) was calculated using Eq. (4). SPSS Statistics (SPSS 25, SPSS Inc., New York, USA) were used for the statistical analysis. The emission factor for tractor exhaust gas under RWE conditions can be analyzed as a ratio, that is, deviation ratio (DR), by dividing the calculated emission factor by the emission factor obtained using the regulation standard, as shown in Eq. (5)^32^. This can provide intuitive results by comparing emission factors calculated according to RWE with emission regulations^33^.

4 $${\text{RSD}}=\frac{S}{Mean}\times 100\left(\%\right),$$

where ${\text{RSD}}$ denotes the relative standard deviation and $S$ denotes standard deviation.

5 $${\text{DR}}=\frac{EF}{{EF}_{s}}\times 100\left(\%\right),$$

where ${\text{DR}}$ denotes the deviation ratio, $EF$ denotes the calculated emission factor (g/kWh), and ${EF}_{s}$ denotes the emission factor from the regulation standard (g/kWh).

## Results

### Engine characteristic profile

**Figure 3:**
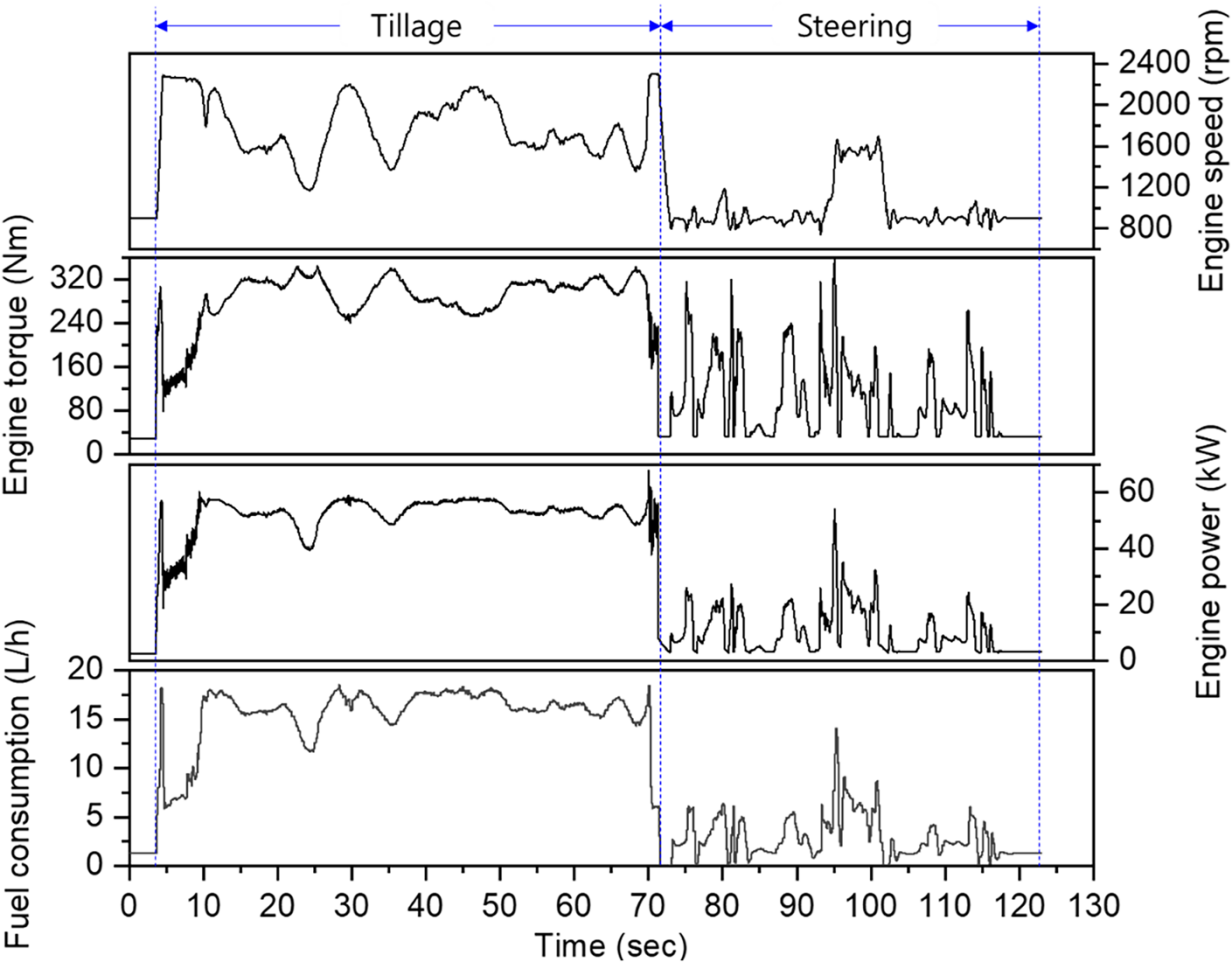
Engine profile of the tractor according to the plow tillage operation.

The profiles of the engine characteristics (rotational speed, torque, power, and fuel consumption) for the tillage and steering sections during plow tillage are shown in Fig. 3. In the tillage section, the engine rotational speed was in the range of approximately 850–2300 rpm, and the engine torque was in the range of approximately 30–350 Nm but had an opposite trend to the engine rotation speed. The engine power was calculated using the engine rotational speed and engine torque, and it showed a large variation in the range of approximately 3–67 kW. In particular, the engine rotation speed and torque exhibited opposite tendencies. This is related to the ability to lower the engine rotation speed and increase the engine torque through throttling down when a high torque is required from the tractor powertrain. This is consistent with the trend in engine characteristics according to the load variation of the tractor during tillage operation, as suggested in a previous study. In addition, fuel consumption was in the range of approximately 6–18 L/h, exhibiting a profile similar to that of engine power^5^. In the steering section, the engine rotation speed and engine torque were 800–1500 rpm and 30–350 Nm, respectively, and the engine power was 3–50 kW and exhibited an irregularly fluctuating profile.

Table 4 shows statistical analysis data of engine characteristic data for each work section according to plow tillage. Overall, higher rotational speed, torque, power, and fuel consumption were observed in the tillage section compared to those in the steering section. In particular, the power in the tillage section was found to be higher than that in the steering section, ranging from approximately 1.1 to 21 times ranges (average 5.3 times). In the RSD, torque and power that are approximately 8.5 and 12.3 times higher, respectively, than those in the tillage section are observed in the steering section. This suggests higher data variability in the steering section compared to the tillage section.

**Table 4:** Statistical description of engine profile according to the plow tillage.

Description		Plow tillage		
		Tillage	Steering	Total
Engine speed (rpm)	Max./Min	2304/1170	1696/802	2304/802
	Avg. ± Std	1743 ± 254	994 ± 229	1446 ± 471
	RSD (%)	14.6	23.0	32.6
Engine torque (Nm)	Max./Min	345/195	359/32.1	359/32.1
	Avg. ± Std	298 ± 26	93.8 ± 68.4	202 ± 112
	RSD (%)	8.6	73.0	55.5
Engine power (kW)	Max./Min	67.0/39.4	54.0/2.8	67.0/2.8
	Avg. ± Std	53.6 ± 3.5	10.0 ± 8.1	33.8 ± 22.2
	RSD (%)	6.6	81.0	65.8
Fuel consumption (L/h)	Max./Min	18.5/9.5	14.1/0.8	18.5/0.8
	Avg. ± Std	16.3 ± 1.4	2.9 ± 2.1	10.0 ± 6.8
	RSD (%)	8.3	73.6	68.0

**Figure 4:**
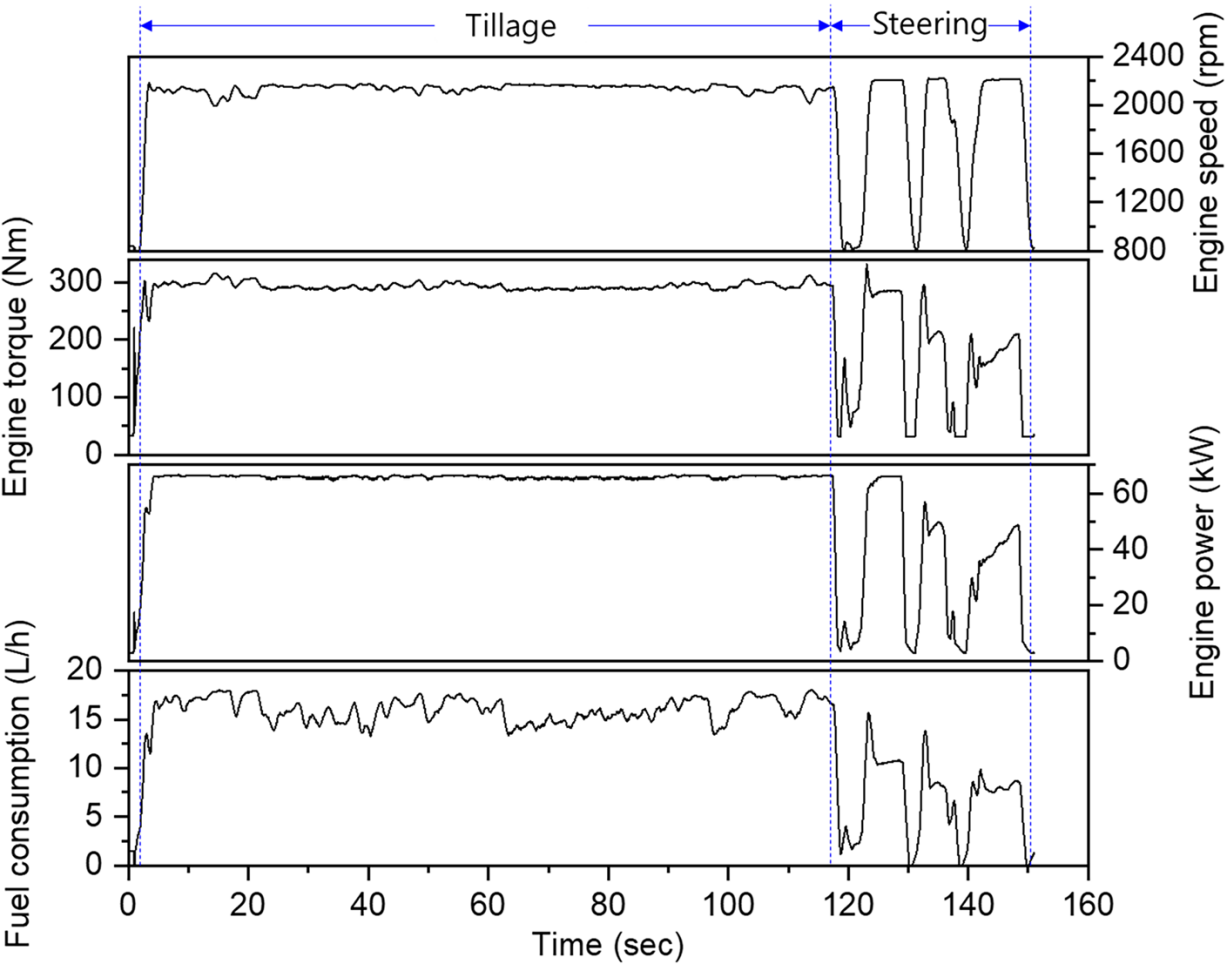
Engine profile of the tractor according to the rotary tillage operation.

Figure 4 shows the engine profile according to the rotary tillage. In the tillage section, the engine rotational speed was in the range of approximately 2000–2200 rpm, with a maximum variability of 10%. The engine torque was in the range of approximately 280–315 Nm and showed fluctuations of up to 13%. The engine output showed a change of up to 5% in the range of approximately 64–67 kW. In addition, the fuel consumption was in the range of approximately 13–18 L/h. In the steering section, the engine rotation speed and torque were 800–2200 rpm and 30–330 Nm, respectively, and the engine power fluctuated irregularly, ranging from 3 to 66 kW. As shown in Fig. 3, engine torque and rotational speed showed very large fluctuations during plow tillage operation but on the other hand, engine performance showed relatively low fluctuations during rotary operation. This is believed to be due to differences in characteristics (particularly, presence or absence of PTO operation) between plow and rotary tillage.

Table 5 shows the statistical analysis results of the engine characteristic data for each work section according to rotary tillage. According to the results, the rotational speed, torque, power, and fuel consumption in the tillage section are higher than those in the steering section, similar to plow tillage. In particular, the power was found to be approximately 177% in the tillage section compared to that in the steering section. The RSDs for the torque and power in the steering section were approximately 2700 and 10,700%, respectively, compared with those in the tillage section.

**Table 5:** Statistical description of engine profile according to the rotary tillage.

Descriptions		Rotary tillage		
		Tillage	Steering	Total
E/G speed (rpm)	Max./Min	2177/1993	2220/800	2220/800
	Avg. ± Std	2139 ± 31	1797 ± 525	2028 ± 342
	RSD (%)	1.5	29.2	16.9
E/G torque (Nm)	Max./Min	315/285	331.4/32	331.4/32
	Avg. ± Std	294 ± 6	180 ± 98	261 ± 73
	RSD (%)	2.0	54.1	28.0
E/G power (kW)	Max./Min	66.6/64.7	66.4/2.8	66.6/2.8
	Avg. ± Std	65.8 ± 0.4	37.2 ± 23.8	57.4 ± 18.4
	RSD (%)	0.6	64.2	32.1
Fuel consumption (L/h)	Max./Min	18.0/13.3	15.6/1.4	18.0/1.4
	Avg. ± Std	15.9 ± 1.2	8.3 ± 5.2	13.9 ± 4.5
	RSD (%)	7.3	62.9	32.1

### Load factor analysis

**Figure 5:**
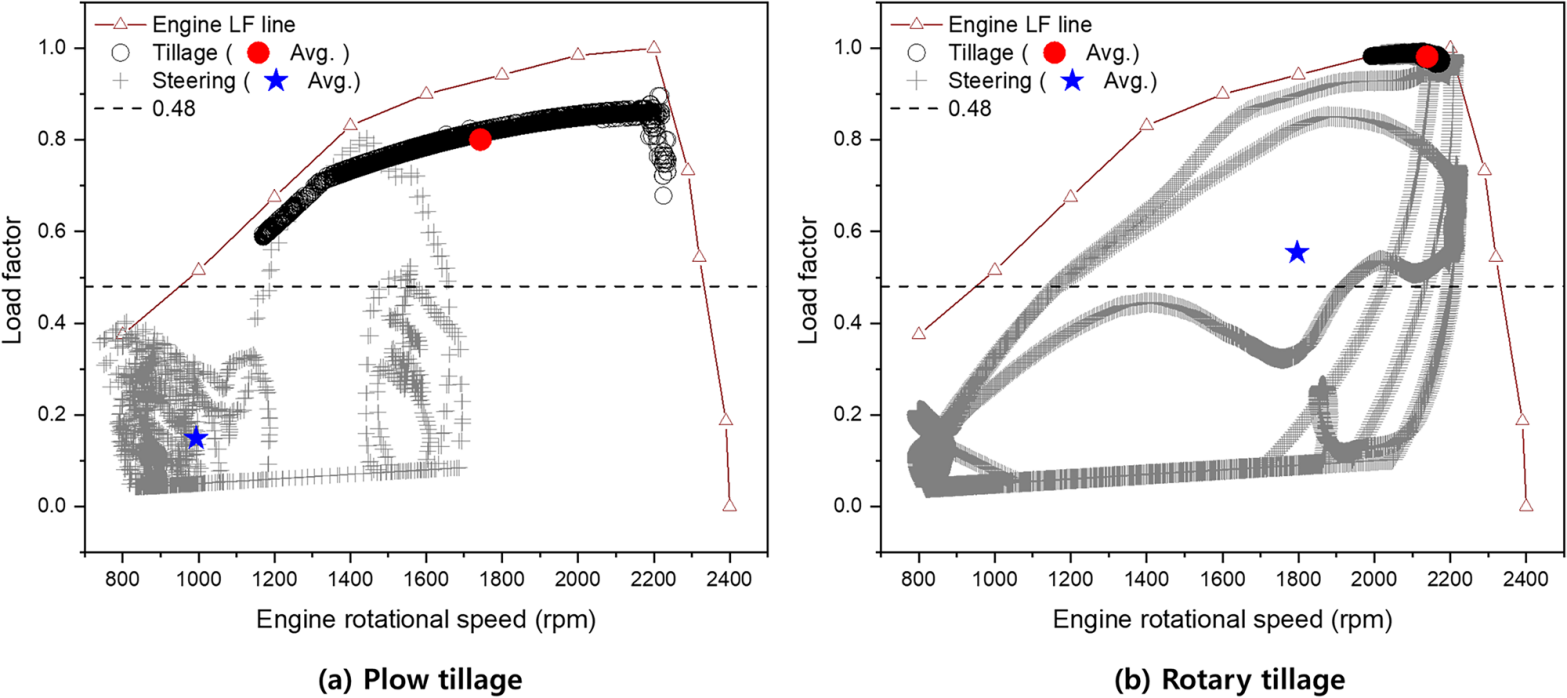
Mapped field-operation data on engine curve (left: plow tillage, right: rotary tillage).

Figure 5 a shows the results of the mapping of plow tillage-section and steering-section data on an engine LF curve. Because this study was performed over a wide range of rotational speeds in the tillage and steering sections during plow tillage, the LFs in the tillage and steering sections are approximately 0.59–0.90 and 0.04–0.8, respectively. Additionally, the average LFs are 0.80 (red circle) and 0.15 (blue star), respectively. This is significantly different from the standard value (0.48), which is collectively applied in current agricultural machinery in Korea regardless of the conditions such as the type of agricultural machinery and working conditions^12^. Figure 5 b shows the results of the mapping of rotary tillage- and steering-section data on an engine LF curve. For rotary tillage, only a relatively narrow rotational speed of 2000–2200 rpm is used in the tillage section, and it can be seen that the operation was performed under a high LF close to the maximum. However, a wide range of rotational speeds is observed in the steering section. The LF in the tillage- and steering-sections are approximately 0.96–0.99 and 0.04–0.99, respectively, and the averaged LFs are 0.98 and 0.55, respectively. This is significantly different from the currently applied LF of 0.48, which is similar to that in the plow tillage case.

### Analysis of the exhaust emission of the tractor due to tillage operations

**Figure 6:**
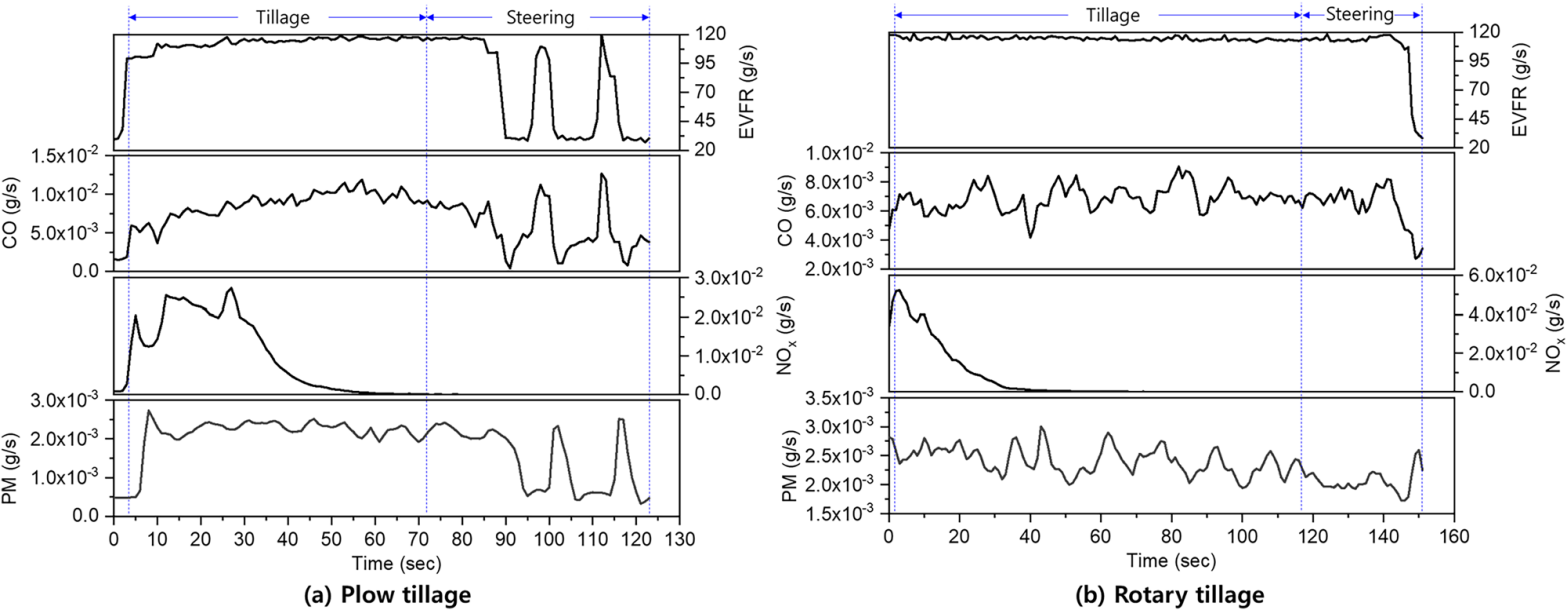
Results of exhaust emission for the tractor engine (left: plow tillage, right: rotary tillage).

The tractor exhaust emissions collected during plow tillage were divided into tillage- and steering-section data, and the results are shown in Fig.6 a. However, it is difficult to distinguish between the tillage- and steering-sections using only the exhaust emission characteristics. Therefore, in this study, the sections of the exhaust emission profile were divided by applying the standard value that divided the work section according to the tractor load characteristics (Figs. 3, 4). The EVFR in the tillage- and steering-sections is in the ranges of approximately 100–120 g/s and 27–120 g/s, respectively. In all sections, the CO and PM emissions fluctuated irregularly, and the NOx emissions fluctuated for 30 s before rising and subsequently decreasing from 30 to 60 s, beyond which they show a tendency of converging at zero. Fig. 6 b shows the tractor exhaust emissions collected during rotary tillage. Similar to plough tillage, the entire section was divided based on the engine characteristics. It can be seen that the EVFR in the tillage and steering sections is in the ranges of approximately 111–120 g/s and 28–118 g/s, respectively. In all sections, the CO and PM fluctuate irregularly, and the NOx emissions fluctuate for 3 s before rising and subsequently decreasing for 3–40 s, beyond which they exhibit a tendency for converging at zero. This trend is similar to that in plow tillage, but the NOx emissions in this case decrease at a faster rate compared to that in plow tillage. In general, exhaust gas emissions are reduced due to the influence of various after-treatment devices, and the tractor used in this study is equipped with SCR, which reduces NOx emissions. As shown in Fig. 6, it can be seen that in both operations, NOx increases at the beginning of the work and then gradually decreases over time, which is believed to be an effect of the operation of the SCR. Overall, the exhaust temperatures during plow tillage and rotary tillage were in the range of 180–191℃ and 192–225℃, respectively. As mentioned earlier, because the exhaust gas temperature measurement location is long enough from the engine, these results are considered to correspond to the research results showing that temperatures above 190°C must be reached for SCR to operate properly^34^.

Based on the plow tillage data, the CO, NOx, and PM emissions were statistically analyzed for the tillage, steering, and entire section (tillage section + steering section), and the results are presented in Table 6. The average CO, NOx, and PM emissions in the tillage section were 8.8 × 10−^3^, 1.0 × 10−^2^, and 2.2 × 10−^3^, respectively, and those in the steering section were 5.6 × 10−^3^, 3.6 × 10−^5^, and 1.4 × 10−^3^, respectively. The RSDs of CO, NOx, and PM were 18.3–52.2, 67.6–151.9%, and 12.6–52.5%, respectively, depending on the section. Consequently, it can be seen that the NOx emissions exhibit the highest fluctuation in the entire section. This is considered to be due to the rapid reduction in the NOx under the influence of SCR after a certain operating time.

**Table 6:** Statistical description of exhaust emissions (CO, NOx, and PM) for tractors based on plow tillage.

Descriptions		Plow tillage		
		Tillage	Steering	Total
EVFR (g/s)	Max./Min	118/100	121/27	121/27
	Avg.$\pm$ Std	114 ± 4.3	69 ± 38.4	95 ± 33.4
CO (g/s)	Max./Min	1.2 × 10^–2^/3.7 × 10^–3^	1.3 × 10^–2^/4.0 × 10^–4^	1.3 × 10^–2^/4.0 × 10^–4^
	Avg. ± Std	8.8 × 10^–3^ ± 1.6 × 10^–3^	5.6 × 10^–3^ ± 2.9 × 10^–3^	7.4 × 10^–3^ ± 2.8 × 10^–3^
NO_x_ (g/s)	Max./Min	2.7 × 10^–2^/4.1 × 10^–8^	1.1 × 10^–4^/3.7 × 10^–8^	2.7 × 10^–2^/3.7 × 10^–8^
	Avg.$\pm$ Std	1.0 × 10^–2^ ± 9.6 × 10^–3^	3.6 × 10^–5^ ± 2.4 × 10^–5^	5.8 × 10^–3^ ± 8.8 × 10^–3^
PM (g/s)	Max./Min	2.7 × 10^–3^/4.9 × 10^–4^	2.5 × 10^–3^/3.2 × 10^–4^	2.7 × 10^–3^/3.2 × 10^–4^
	Avg.$\pm$ Std	2.2 × 10^–3^ ± 2.8 × 10^–4^	1.4 × 10^–3^ ± 7.6 × 10^–4^	1.9 × 10^–3^ ± 6.6 × 10^–4^

The rotary tillage data were statistically analyzed based on the CO, NOx, and PM emissions in each section, as shown in Table 7. The average CO, NOx, and PM emissions in the tillage section are 6.9×10^−3^, 4.3×10^−3^, and 2.4×10^−3^, respectively, and those in the steering section are 6.5×10^−3^, 2.9×10^−5^, and 2.1×10^−3^, respectively.

**Table 7:** Statistical description of exhaust emissions (CO, NOx, and PM) of tractors based on rotary tillage.

Descriptions		Rotary tillage		
		Tillage	Steering	Total
EVFR (g/s)	Max./Min	120/111	118/28	120/28
	Avg.$\pm$ Std	114 $\pm$ 1.3	107 $\pm$ 22	112 $\pm$ 12
CO (g/s)	Max./Min	9.1 × 10^–3^/4.2 × 10^–3^	8.2 × 10^–3^/2.7 × 10^–3^	9.1 × 10^–3^/2.7 × 10^–3^
	Avg. ± Std	6.9 × 10^–3^ ± 8.6 × 10^–4^	6.5 × 10^–3^ ± 1.2 × 10^–3^	6.8 × 10^–3^ ± 9.9 × 10^–4^
NOx (g/s)	Max./Min	4.0 × 10^–2^/2.7 × 10^–9^	8.0 × 10^–5^/4.9 × 10^–9^	4.0 × 10^–2^/2.7 × 10^–9^
	Avg.$\pm$ Std	4.3 × 10^–3^ ± 9.0 × 10^–3^	2.9 × 10^–5^ ± 1.8 × 10^–5^	3.1 × 10^–3^ ± 7.9 × 10^–3^
PM (g/s)	Max./Min	3.0 × 10^–3^/1.9 × 10^–3^	2.6 × 10^–3^/1.7 × 10^–3^	3.0 × 10^–3^/1.7 × 10^–3^
	Avg.$\pm$ Std	2.4 × 10^–3^ ± 2.3 × 10^–4^	2.1 × 10^–3^ ± 1.9 × 10^–4^	2.3 × 10^–3^ ± 2.6 × 10^–4^

### Analysis of emission factors for the tractor by air pollutants

**Figure 7:**
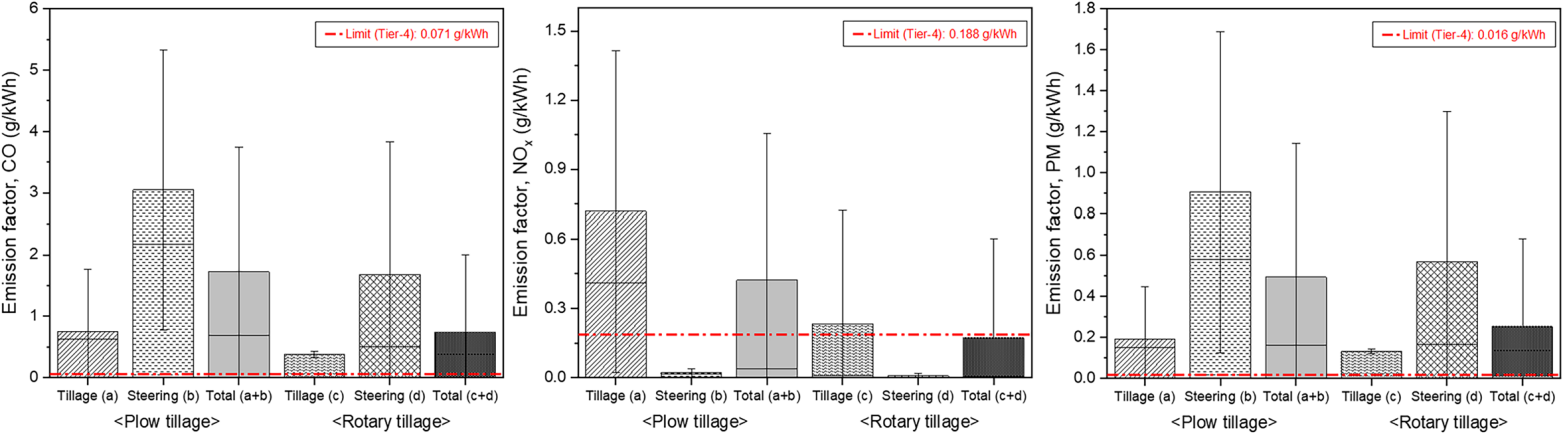
Results of analysis of emission factors for tractor engines and comparison with emission regulation stage (left: CO, middle: NO_x_, right: PM).

The emission factors for each working condition based on the obtained tractor emissions (CO, NOx and PM) and LFs were calculated and compared with those outlined in the Tier-4 emission standards, as shown in Fig. 7. When compared with the Tier-4 standard, the emission factors of CO are higher under all conditions, as shown in Fig. 7 a. The emission factors for NOx show similar or higher values in all conditions except the steering section for both tillage operations when compared to Tier-4 emission standards, as shown in Fig. 7 b. The emission factor for PM under all conditions showed higher than the Tier-4 emission standard, as shown in Fig. 7 c.

Table 8 presents the analysis results of the average emission factor for each working condition. The CO values during plow tillage are 0.754, 3.046, and 1.725 g/kWh in the tillage, steering, and total sections, respectively, and those during rotary tillage are 0.378, 1.682, and 0.735 g/kWh, respectively. The NOx emissions during plow tillage are 0.718, 0.021, and 0.423 g/kWh in the tillage, steering, and total sections, respectively, and those during rotary tillage are 0.232, 0.007, and 0.171 g/kWh, respectively. The PM emissions during plough tillage are 0.191, 0.906, and 0.494 g/kWh in the tillage, steering, and total sections, respectively, and those during rotary tillage are 0.132, 0.570, and 0.252 g/kWh, respectively.

**Table 8:** Results of analysis of average emission factor for each working condition.

Operations	Conditions	Averaged emission factor (g/kWh)		
		CO	NO_x_	PM
Plow tillage	Tillage (a)	0.754	0.718	0.191
	Steering (b)	3.046	0.021	0.906
	Total (a + b)	1.725	0.423	0.494
Rotary tillage	Tillage (c)	0.378	0.232	0.132
	Steering (b)	1.682	0.007	0.570
	Total (c + d)	0.735	0.171	0.252

### Evaluation of emission factors for each working condition using emission standard

**Figure 8:**
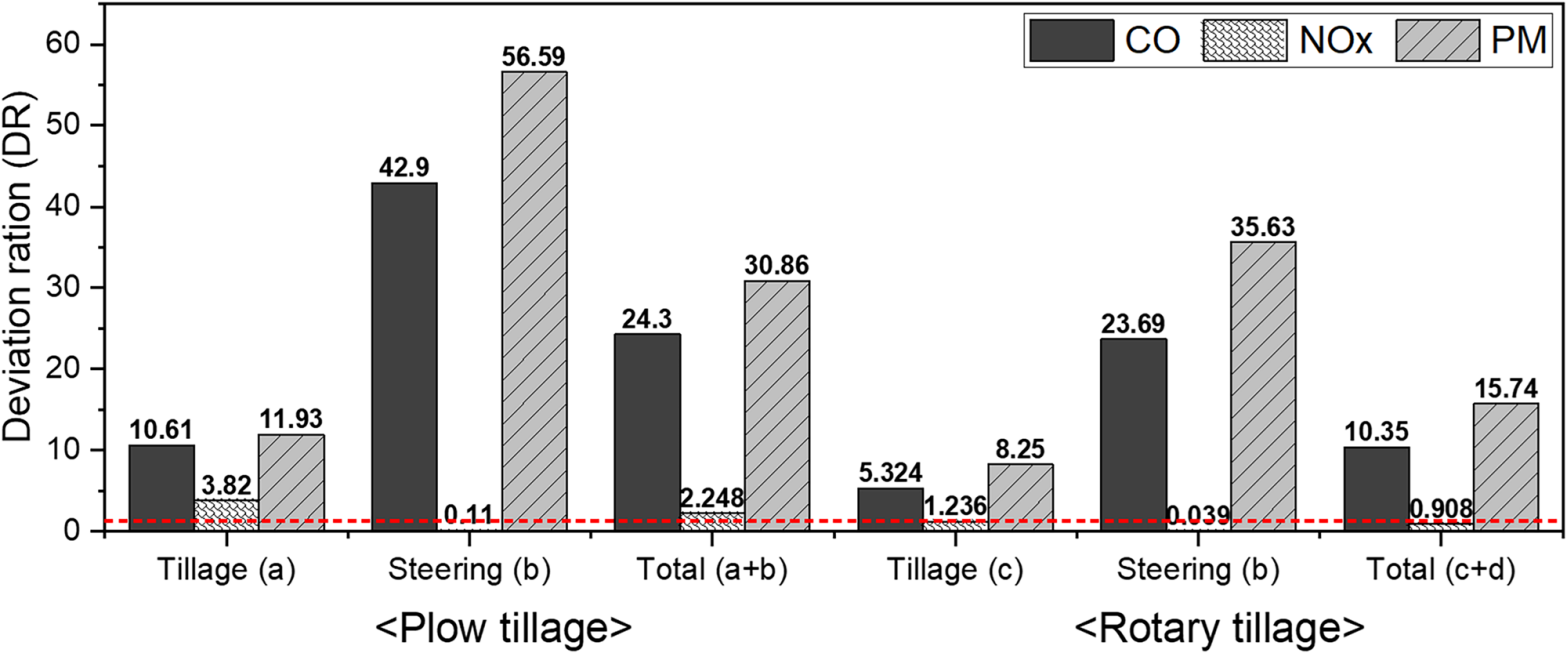
Comparison of deviation ratios between emission factors for each condition derived from this study and regulations.

The DR was evaluated by comparing the emission factors for each analyzed working condition (Table 8) with Tier-4 emission regulations, as shown in Fig. 8. DR is a numerical value that indicates how much higher the measured emission factor under each condition is compared to the reference value, thereby enabling an intuitive comparison. The measured DR of CO was found to be higher than 1 in all operating conditions, which indicates that the measured emission factor is higher than the Tier-4 emission factor. The overall measured emission factor of CO was found to be 5.324 to 42.9 times higher than the Tier-4 emission factor. The minimum value of this difference was 5.324 times in the tillage section (c) for the rotary tillage, and the maximum value was 42.9 times in the tillage section (b) for plow tillage. The measured DR of NOx was found to be less than 1 in the total conditions of the steering for plow tillage, and steering and total for rotary tillage, showing that it satisfies Tier-4 emission standards. This result is due to the fact that the NOx emissions in the steering section are close to zero. In three working conditions other than those previously mentioned, the DR of NOx ranged from 1.236 to 3.82, exceeding Tier-4 emission standards. In all six conditions, the DR of PM was found to exceed 1, which was higher than the Tier-4 emission standard, and the DR was found to be 8.25–56.59, which was very high compared to the Tier-4 emission standard.

## Discussions

The aim of this study is to measure the LF and emissions of tractors under actual working conditions and evaluate the emission factor based on LF and emission. The proposed PEMS-based measurement system was considered to be suitable for collecting exhaust emissions in the field. Based on this measurement system, exhaust emission was measured in the field, and data analysis by tillage and steering sections were analyzed. The LF value according to engine rotational speed was mapped to the engine performance map and compared with the current standard value of 0.48. In this study, the emission factor was analyzed based on LF and emission data measured under actual working conditions. It was concluded that the emission factor shows a significant difference when compared to the Tier-4 emission standard. This difference can be considered a reasonable result since the Tier-4 emission standards are not derived from actual operating conditions in the field. Nevertheless, to verify the results of this study, the results of this study were compared with similar previous studies. Data related to agricultural machinery types, power, emission standards, and exhaust emission (CO, NOx and PM) by operation derived from previous research are listed in Table 9. The subjects of investigation for comparative analysis are 70–132 kW tractors and 86 kW agricultural combine harvester. In previous studies, CO overall ranged from 0.2 to 5.8 g/kWh, and the values proposed in this study (plow tillage: 1.725 g/kWh and rotary tillage: 0.735 g/kWh) are within the range suggested in previous studies. In previous studies, NOx was found to be in the overall range of 2.06 to 10.6 g/kWh, which is much higher than the 0.171 to 0.423 g/kWh data analyzed in this study. This is presumed to be because the tractor used in this study was equipped with an SCR, which reduced NOx emissions. In the case of PM, in previous studies, it was found to be in the range of 0.007–0.0.89 g/kWh, and in this study, it was found to be in the range of 0.252–0.494 g/kWh. It is believed that the wide range of PM emission factor is because the load appears differently depending on the various tasks performed by the tractor. It was found to be as low as 0.007 g/kWh during transport work under low-load under on-road condition during tractor work, and as high as 0.89 g/kWh during high-load work such as cultivation under off-road condition. Thus, this can be considered a somewhat reasonable difference considering the irregular variability of field work. As a result, the reasonableness of the actual operation-based emission factor derived in this study was evaluated by comparing it with previous studies.

**Table 9:** Comparison of measured emission factor for agricultural machinery in actual working condition.

Item	Type	Power (kW)	Operation	Averaged emission factor (g/kWh)			Reference
				CO	NOx	PM	
This study	Tractor	67	Plow tillage	1.725	0.423	0.494	–
			Rotary tillage	0.735	0.171	0.252	
Previous study		70	Harrowing	1.45	8.89	–	^36^
			Stubble cultivation	1.1	6.54	–	
			Transport (8.8t)	1.37	8.20	–	
			Transport (12t)	2.07	10.6	–	
			Plow tillage	1.07	8.84	–	
		82	Rotary harrowing	0.20	4.84		^37^
		112	Cultivation	2.01	2.92	0.89	^38^
		132	Transport (4t)	0.72	1.44	0.007	^11^
	Combine harvester	86	Idling	1.88	2.06	0.0375	^39^
			Moving	2.96	4.86	0.2070	
			Working	5.80	6.36	0.3453	

## Conclusions

In this study, a method for measuring the LFs and tractor exhaust emissions during actual tillage operations using a PEMS and calculation of the emission factors based on various evaluation methods is provided. A comparison of the measured emission factors with the Tier-4 emission standard are also included in the proposed method.

The tractor emission measurement system was built using a PEMS and GPS to measure the exhaust gas, and the ECU data were collected through CAN communication to record information on the engine operation. Data were collected from plow tillage and rotary tillage operations in a paddy field in Korea, wherein the tractor engine characteristics (torque and rotational speed) were significantly different under each working condition. This had a direct effect on the engine LF characteristics, and caused the LF calculated in this study to be significantly different from the current applied value of 0.48. Additionally, the engine LFs for the tillage and steering sections were mapped to the engine curve for each operation to assist in the determination of the statistical descriptions of the engine characteristics and exhaust emissions. Based on the results, the exhaust emissions showed a tendency to significantly fluctuate according to the characteristics of the working condition, but did not exhibit a linearity that immediately changes based on changes in the engine characteristics. Moreover, the measured emission factor was compared with the emission limit and a numerical value was obtained. The measured value was higher than the standard emission factor (Tier-4) under various conditions. Although the emission factor measured in this study was higher than the standard emission factor, it cannot be considered inappropriate. This difference is considered to be due to fact that emission standards are typically measured using an engine dynamometer^35^.

The results of this study can be useful because they suggest a range of emission factors for exhaust gases generated during actual agricultural works. However, this study has several limitations. First, information on the triggers of the SCR operation is not provided, and only three of the various air pollutants (CO, NOx, and PM) are targeted. Second, the because experiment was conducted at only one site (paddy field) in Korea and only one tractor model was used, data is not enough. However, despite these limitations, this study can serve as a reference for the measurement of exhaust emissions and evaluation of the emission factors because it presents both the emissions and emission factors as quantitative values ​​obtained by attaching a PEMS to a tractor, which is a suitable representative of the various types of agricultural machinery. It is noteworthy that the obtained results are somewhat different from Tier-4 emission standard. In addition, because the method in this study is based on exhaust emissions emitted under actual working conditions, the effect on the actual atmospheric environment can be directly confirmed. Therefore, it is expected that this method will assist in the accurate quantification of national air pollutant emissions, thereby contributing to the construction of the national air pollutant inventory.

In future research, we plan to collect tractor emission data considering the engine temperature (cold and hot), whether the SCR is in operation, various working type (idling, seedling, etc), soil conditions, and different implement types. Furthermore, reliable research on tractor exhaust gas emissions and emission factors can be conducted by establishing a database that considers various operating conditions.

## Data Availability

The data for this manuscript are not publicly available but may be accessed upon request to the corresponding author.

## References

[1] Yeo, S. J., Kim, J. & Lee, W. J. Potential economic and environmental advantages of liquid petroleum gas as a marine fuel through analysis of registered ships in South Korea. *J. Clean. Prod.*10.1016/j.jclepro.2021.129955 (2022). 10.1016/j.jclepro.2021.129955

[2] Hou, X., Xu, C., Li, J., Liu, S. & Zhang, X. Evaluating agricultural tractors emissions using remote monitoring and emission tests in Beijing China. *Biosyst. Eng.* **213**, 105–118. 10.1016/j.biosystemseng.2021.11.017 (2022). 10.1016/j.biosystemseng.2021.11.017

[3] NAIR (National Air Emission Inventory and Research), Handbook of estimation methods for national air pollutant emissions (IV). (2020)

[4] NAIR (National Air Emission Inventory and Research), 2017 National air pollutant emissions. (2020).

[5] Kim, W. S., Kim, Y. J., Park, S. U. & Kim, Y. S. Influence of soil moisture content on the traction performance of a 78-kW agricultural tractor during plow tillage. *Soil Tillage Res.*10.1016/j.still.2020.104851 (2020). 10.1016/j.still.2020.104851

[6] KAMICO, KSAM, Agricultural machinery yearbook in Republic of Korea, Korea agricultural machinery industry cooperative and Korean society for agricultural machinery. (2021).

[7] Kim, J. T., Im, D., Cho, S. J. & Park, Y. J. A study on the prediction of driving performance of agricultural tractors driving on dry sand. *J. Biosyst. Eng.* **47**, 502–509. 10.1007/s42853-022-00164-8 (2022). 10.1007/s42853-022-00164-8

[8] Koo, Y. M. PTO torque and draft analyses of an integrated tractor-mounted implement for round ridge preparation. *J. Biosyst. Eng.* **47**, 330–343. 10.1007/s42853-022-00146-w (2022). 10.1007/s42853-022-00146-w

[9] Rahmanian-Koushkaki, H., Mahmoodi-Eshkaftaki, M. & Azimi-Nejadian, H. Simulation of draught force during chisel ploughing using discrete element method. *J. Biosyst. Eng.* **47**, 152–166. 10.1007/s42853-022-00133-1 (2022). 10.1007/s42853-022-00133-1

[10] Baek, S. M. *et al.* A study on the emissions of SO_x_ and NH3 for a 78 kW class agricultural tractor according to agricultural operations. *Korean J. Agric. Sci.* **47**(4), 1135–1145. 10.7744/kjoas.20200095 (2020). 10.7744/kjoas.20200095

[11] Lijewski, P. & Merkisz, J. Exhaust emissions from farm tractors operating in urban areas. *WIT Trans. Built Environ.* **130**, 447–453. 10.2495/UT130351 (2013). 10.2495/UT130351

[12] Kim, M. S. *et al.* Analysis of emission characteristics and estimation of air pollutants emitted from small ship. *J. Korean Soc. Atmos. Environ.* **38**, 258–268. 10.5572/KOSAE.2022.38.2.258 (2022). 10.5572/KOSAE.2022.38.2.258

[13] Giechaskiel, B. *et al.* Framework for the assessment of PEMS (portable emissions measurement systems) uncertainty. *Environ. Res.* **166**, 251–260. 10.1016/j.envres.2018.06.012 (2018). 29908456 10.1016/j.envres.2018.06.012 PMC6143386

[14] Triantafyllopoulos, G. *et al.* A study on the CO_2_ and NO_x_ emissions performance of Euro 6 diesel vehicles under various chassis dynamometer and on-road conditions including latest regulatory provisions. *Sci. Total Environ.* **666**, 337–346. 10.1016/j.scitotenv.2019.02.144 (2019). 30798242 10.1016/j.scitotenv.2019.02.144

[15] Xie, H. *et al.* Parallel attention-based LSTM for building a prediction model of vehicle emissions using PEMS and OBD. *Measurement*10.1016/j.measurement.2021.110074 (2021). 10.1016/j.measurement.2021.110074

[16] Kim, S. H. & Lee, D. W. Experimental research for CO2 emission estimation of medium-scale excavator reflecting work characteristics. *J. Korean Soc. Civ. Eng.* **37**, 717–727. 10.12652/Ksce.2017.37.4.0717 (2017). 10.12652/Ksce.2017.37.4.0717

[17] Qi, L. *et al.* Intermediate-volatility organic compound emissions from nonroad construction machinery under different operation modes. *Environ. Sci. Technol.* **53**, 13832–13840. 10.1021/acs.est.9b01316 (2019). 31691567 10.1021/acs.est.9b01316

[18] Tan, D. *et al.* Study on real-world power-based emission factors from typical construction machinery. *Sci. Total Environ.*10.1016/j.scitotenv.2021.149436 (2021). 34365269 10.1016/j.scitotenv.2021.149436

[19] Tu, R. *et al.* Real-world emissions of construction mobile machines and comparison to a non-road emission model. *Sci. Total Environ.*10.1016/j.scitotenv.2021.145365 (2021). 33736176 10.1016/j.scitotenv.2021.145365

[20] Zhang, Q. *et al.* Emission characteristics and chemical composition of particulate matter emitted by typical non-road construction machinery. *Atmos. Pollut. Res.* **11**, 679–685. 10.1016/j.apr.2019.12.018 (2020). 10.1016/j.apr.2019.12.018

[21] Merkisz, J., Lijewski, P., Fuc, P., Siedlecki, M. & Weymann, S. The use of the PEMS equipment for the assessment of farm fieldwork energy consumption. *Appl. Eng. Agric.* **31**, 875–879. 10.13031/aea.31.11225 (2015). 10.13031/aea.31.11225

[22] Lindgren, M. & Hansson, P. A. Effects of engine control strategies and transmission characteristics on the exhaust gas emissions from an agricultural tractor. *Biosyst. Eng.* **83**(1), 55–65. 10.1006/bioe.2002.0099 (2002). 10.1006/bioe.2002.0099

[23] Shin, C. S., Park, T., Hong, D. H. & Kim, T. H. Analysis of air pollutant emissions from agricultural machinery in South Korea. *Korean Soc. Manuf. Process Eng.* **18**, 14–25. 10.14775/ksmpe.2019.18.3.014 (2019). 10.14775/ksmpe.2019.18.3.014

[24] Suarez-Bertoa, R. *et al.* On-road measurement of NH3 and N2O emissions from a Euro V heavy-duty vehicle. *Atmos. Environ.* **139**, 167–175. 10.1016/j.atmosenv.2016.04.035 (2016). 10.1016/j.atmosenv.2016.04.035

[25] Lee, J. H. *et al.* Analysis of emission of agricultural tractor according to engine load factor during tillage operation. *J. Drive Control* **19**(4), 54–61. 10.7839/ksfc.2022.19.4.054 (2022). 10.7839/ksfc.2022.19.4.054

[26] Baek, S. Y. *et al.* Design verification of an E-driving system of a 44 kW-class electric tractor using agricultural workload data. *J. Drive Control* **19**(4), 36–45. 10.7839/ksfc.2022.19.4.036 (2022). 10.7839/ksfc.2022.19.4.036

[27] Drozd, G. T. *et al.* Time resolved measurements of speciated tailpipe emissions from motor vehicles: Trends with emission control technology, cold start effects, and speciation. *Environ. Sci.Technol.* **50**(24), 13592–13599. 10.1021/acs.est.6b04513 (2016). 27993057 10.1021/acs.est.6b04513

[28] Zare, A. *et al.* Analysis of cold-start NO2 and NOx emissions, and the NO2/NOx ratio in a diesel engine powered with different diesel-biodiesel blends. *Environ. Pollut.*10.1016/j.envpol.2021.118052 (2021). 34479164 10.1016/j.envpol.2021.118052

[29] Han, X., Kim, H. J., Jeon, C. W. & Kim, J. H. Simulation study to develop implement control and headland turning algorithms for autonomous tillage operations. *J. Biosyst. Eng.* **44**, 245–257. 10.1007/s42853-019-00035-9 (2019). 10.1007/s42853-019-00035-9

[30] Baek, S. M. *et al.* Analysis of engine load factor for a 78 kW class agricultural tractor according to agricultural operations. *J. Drive Control* **19**(1), 16–25. 10.7839/ksfc.2022.19.1.016 (2022). 10.7839/ksfc.2022.19.1.016

[31] NAS (National Institute of Agricultural Sciences), 2019 agricultural machinery utilization survey. (2020).

[32] Oak, S. *et al.* Characteristics of real-road driving NOx emissions from Korean light-duty vehicles regarding driving routes. *Trans. KSAE.* **23**(1), 130–213. 10.7467/KSAE.2015.23.1.130 (2015). 10.7467/KSAE.2015.23.1.130

[33] Zare, A. *et al.* Hazardous particles during diesel engine cold-start and warm-up: Characterisation of particulate mass and number under the impact of biofuel and lubricating oil. *J. Hazard. Mater.*10.1016/j.jhazmat.2023.132516 (2023). 37703733 10.1016/j.jhazmat.2023.132516

[34] Slimarik, D., Polcar, A., & Jukl, M., Influence of exhaust gas temperature on treatment of harmful pollutants. *MendelNet 2014 Conference* (2014).

[35] Park, J., Lee, J., Kim, S., Kim, J. & Ahn, K. A study on the emission characteristics of Korean light-duty vehicles in real-road driving conditions. *Trans. KSAE.* **21**(6), 123–134. 10.7467/KSAE.2013.21.6.12 (2013). 10.7467/KSAE.2013.21.6.12

[36] Hansson, P. A., Norén, O. & Bohm, M. Effects of specific operational weighting factors on standardized measurement of tractor engine emissions. *J. Agric. Eng. Res.* **74**(4), 347–353. 10.1006/jaer.1999.0471 (1999). 10.1006/jaer.1999.0471

[37] Lovarelli, D. & Bacenetti, J. Bridging the gap between reliable data collection and the environmental impact for mechanised field operation. *Biosyst. Eng.* **160**, 109–123. 10.1016/j.biosystemseng (2017). 10.1016/j.biosystemseng

[38] Merkisz, J., LiJewski, P., Fuć, P. & Weymann, S. Exhaust emission tests from non-road vehicles conducted with the use of PEMS analyzers. *Eksploatacja i Niezawodność* **15**(4), 364–368 (2013).

[39] Hou, X. *et al.* Emission inventory research of typical agricultural machinery in Beijing, China. In *Atmospheric Environment* (eds Hou, X. *et al.*) (Elsevier, 2019).

